# ﻿Five new epiphytic species of *Vishniacozyma* (Bulleribasidiaceae, Tremellales) from China

**DOI:** 10.3897/mycokeys.113.140598

**Published:** 2025-02-12

**Authors:** Shan Liu, Dan-Yang Cai, Chun-Yue Chai, Feng-Li Hui

**Affiliations:** 1 School of Life Science, Nanyang Normal University, Nanyang 473061, China Nanyang Normal University Nanyang China; 2 Research Center of Henan Provincial Agricultural Biomass Resource Engineering and Technology, Nanyang Normal University, Nanyang 473061, China Nanyang Normal University Nanyang China

**Keywords:** Basidiomycota, new species, phylloplane, phylogenetic analysis, taxonomy

## Abstract

The genus *Vishniacozyma*, globally distributed, encompasses numerous epiphytic and endophytic species. In this study, five new species are proposed to accommodate eleven yeast strains isolated from leaves of different plants: *V.diospyri***sp. nov.** (holotype CICC 33574^T^), *V.guiyangensis***sp. nov.** (holotype CICC 33569^T^), *V.pingtangensis***sp. nov.** (holotype CICC 33596^T^), *V.eriobotryae***sp. nov.** (holotype GDMCC 2.312^T^), and *V.tianchiensis***sp. nov.** (holotype CICC 33617^T^) using phenotypic and phylogenetic characters. Phylogenetic analysis was based on the internal transcribed spacer (ITS) region and the D1/D2 domain of the large subunit (LSU) rRNA gene. Illustrations and descriptions of these five taxa are provided, along with comparative analyses with closely related species within the genus. This research highlights the considerable diversity of *Vishniacozyma* species in China and contributes valuable data for future investigations in fungal systematics and evolution.

## ﻿Introduction

The genus *Vishniacozyma*, typified by *V.carnescens* (Verona & Luchetti) X.Z. Liu, F.Y. Bai, M. Groenew. & Boekhout, was established by Liu et al. (2015) based on results of their seven-marker phylogeny. In this study, 11 previously described species in the genera *Bullera*, *Cryptococcus*, and *Trimorphomyces* were transferred to *Vishniacozyma*. Subsequently, 21 new species, including *V.ellesmerensis* M. Tsuji, Y. Tanabe, W.F. Vincent & Mas (Tsuji et al. 2019), *V.changhuana* C.F. Lee & Chin F. Chang, *V.taiwanica* C.F. Lee & Chin F. Chang (Chang et al. 2019), *V.kurtzmanii* Yurkov (Yurkov and Kurtzman 2019), *V.alagoana* C.R. Félix, D.A. Andrade, J.H. Almeida, H.M. Navarro, Fell & Landell (Félix et al. 2020), *V.phoenicis* Kachalkin, A.S. Venzhik & Tomash (Crous et al. 2020), *V.europaea* Q.M. Wang, F.Y. Bai & A.H. Li, *V.melezitolytica* Q.M. Wang, F.Y. Bai & A.H. Li, *V.pseudopenaeus* Q.M. Wang, F.Y. Bai & A.H. Li (Li et al. 2020), *V.insularis* Y.P. Tan, Marney & R.G. Shivas (Tan et al. 2021), *V.pseudodimennae* X.Z. Liu, F.Y. Bai & X.Y. Wei (Wei et al. 2022), *V.terrae* Y. Park, Maeng & S. Sriniv (Maeng et al. 2023), *V.pseudocarnescens* H.Y. Zhu, X.Z. Liu & F.Y. Bai (Zhu et al. 2023), *V.catalpae* Q.M. Wang, *V.marinae* Q.M. Wang, *V.paravictoriae* Q.M. Wang, *V.pini* Q.M. Wang, *V.pyri* Q.M. Wang, *V.sinopodophylli* Q.M. Wang, *V.zhenxiongensis* Q.M. Wang (Jiang et al. 2024), and *V.floricola* Péter, Álvarez-Pérez & Dlauchy (Dlauchy et al. 2024) have been accepted in the genus.

To date, the sexual state of *Vishniacozyma* is only known in *V.nebularis* (Vishniac) A.M. Yurkov, which was found on a dead branch from Taiwan (Kirschner and Chen 2008). The remaining representatives of the genus are asexual morphs resembling yeast stages from the genus *Cryptococcus* and reproduce via budding. Some species may form ballistoconidia and poorly developed pseudohyphae (Liu et al. 2015). Physiologically, the members of the genus lack fermentative ability, possess CoQ-9 or Q-10 as a predominant ubiquinone, and assimilate various carbon sources, but not methanol and hexadecane (Liu et al. 2015; Zhu et al. 2023).

*Vishniacozyma* has a crucial role in maintaining forest biodiversity, so some species, such as *V.changhuana* and *V.taiwanica*, are abundant in the mangrove ecosystem (Chang et al. 2019; Nimsi et al. 2023). Besides ecological functions, biocontrol, biotechnological, and medicinal values of this genus are also notable. For instance, *V.foliicola* (Q.M. Wang & F.Y. Bai) A.M. Yurkov and *V.victoriae* (M.J. Montes, Belloch, Galiana, M.D. García, C. Andrés, S. Ferrer, Torr.-Rodr. & J. Guinea) X.Z. Liu, F.Y. Bai, M. Groenew. & Boekhout, as endophytes, inhibit the growth of phytopathogenic fungi to control postharvest diseases in stored fruit (Bilański and Kowalski 2022; Gorordo et al. 2022; Nian et al. 2023). *V.psychrotolerans* (V. de García, Zalar, Brizzio, Gunde-Cim. & van Broock) A.M. Yurkov can accumulate lipids for biodiesel production (Deeba et al. 2017, 2018). *V.victoriae* is a suitable candidate for the production of fatty acids and ergosterol at the industrial scale (Villarreal et al. 2018).

*Vishniacozyma* is a ubiquitous genus, widely distributed in subtropical, tropical, temperate, and even cold regions, including America, Asia, Australia, and Europe (Montes et al. 1999; Vishniac 2002; de Garcia et al. 2012; Tsuji et al. 2019; Yurkov and Kurtzman 2019; Crous et al. 2020; Félix et al. 2020; Li et al. 2020; Tan et al. 2021; Zhu et al. 2023; Jiang et al. 2024). *Vishniacozyma* species are mainly endophytes, epiphytes, and saprophytes of plants, especially of the leaves (Wang et al. 2011; Félix et al. 2020; Li et al. 2020; Tan et al. 2021; Jiang et al. 2024), but they can also grow across diverse terrestrial environments, including soil (Li et al. 2020; Maeng et al. 2023; Jiang et al. 2024), air inside caves (Ogórek et al. 2021), and dogs (Harvey et al. 2023).

*Vishniacozyma* seems to be a very diversified genus in China, since of the 32 species included in them, 15 have been described from this country (Kirschner and Chen 2008; Wang et al. 2011; Chang et al. 2019; Li et al. 2020; Wei et al. 2022; Zhu et al. 2023; Jiang et al. 2024), but to know the true richness of *Vishniacozyma* species, more studies are necessary in different areas. The aim of this study is to contribute to knowledge of this genus, based on eleven strains of tremellomycetous fungi isolated from different areas across China. According to molecular (ITS and LSU) and phenotypic analyses, they were identified as five new species of *Vishniacozyma* that are described and illustrated in this paper.

## ﻿Materials and methods

### ﻿Sample collection and yeast isolation

Samples were collected from Guizhou and Henan Provinces of China. Yeast strains were isolated from leaf surfaces using the improved ballistospore-fall method as described by Nakase and Takashima (1993). Specifically, the fresh and healthy leaves were sectioned into small pieces and attached to the inner lid of a Petri dish using a thin layer of petroleum jelly. The dish contained yeast extract-malt extract (YM) agar (0.3% yeast extract, 0.3% malt extract, 0.5% peptone, 1% glucose, and 2% agar) with supplemented 0.01% chloramphenicol to limit bacterial growth. Plates were incubated at 20 °C and monitored daily for the presence of colonies, which were selected and purified by streaking them on separate YM agar plates. Following purification, yeast strains were suspended in 20% (v/v) glycerol and stored at −80 °C. Cultures of all isolates were preserved at the Microbiology Lab, Nanyang Normal University, Henan, China, cultures type are preserved as a metabolically inactive state in the CICC (China Centre of Industrial Culture Collection, Beijing, PR China) and cultures ex-type in the PYCC (Portuguese Yeast Culture Collection, Caparica, Portugal).

### ﻿Phenotypic examination

Morphological characterization and physiological and biochemical tests were carried out according to standard methods described by Kurtzman et al. (2011). The potential sexual reproduction in new species was assessed on corn meal agar (CMA: 2.5% corn starch and 2% agar), potato dextrose agar (PDA: 20% potato infusion, 2% glucose, and 2% agar), and V8 agar (10% V8 juice and 2% agar) (Li et al. 2020). A loopful of cells of each test strain is mixed on an agar plate incubated at 17 °C for up to eight weeks. The ballistoconidium-forming activity of all new species was observed by the inverted-plate method (do Carmo-Sousa and Phaff 1962) using CMA at 17 °C. Glucose fermentation tests were carried out in a liquid medium using Durham fermentation tubes. Assimilation of carbon and nitrogen compounds was examined in a liquid medium, and starved inoculum was employed for the nitrogen test (Kurtzman et al. 2011). Growth at different temperatures (15, 20, 25, 30, 35, and 37 °C) was determined by cultivation on YM agar. Cell morphology was examined using LEICA DM2500 cameras (LEICA, Wetzlar, Germany) and LAS V4.13 software. All novel taxonomic descriptions and proposed names were deposited in the MycoBank database (http://www.mycobank.org).

### ﻿DNA extraction, PCR, and sequencing

The genomic DNA was extracted from yeast strains using the Ezup Column Yeast Genomic DNA Purification Kit, according to the manufacturer’s directions (Sangon Biotech Co., Shanghai, China). The ITS region and the D1/D2 domain of the LSU rRNA gene were amplified using the ITS1/ITS4 (White et al. 1990) and NL1/NL4 (Kurtzman and Robnett 1998) primers, respectively. The amplifications were conducted in a 25 µL reaction-volume tube containing 9.5 µL of ddH_2_O, 12.5 µL of 2× Taq PCR Master Mix with blue dye (Sangon Biotech Co., Shanghai, China), 1 µL of DNA template, and 1 µL of each primer. The following parameters were used to amplify the ITS and D1/D2 regions: an initial denaturation step of 2 min at 95 °C, followed by 35 cycles of 30 s at 95 °C, 30 s at 51 °C, 40 s at 72 °C, and a final extension of 10 min at 72 °C (Toome et al. 2013). The PCR products were purified and sequenced by Sangon Biotech Co., Ltd. (Shanghai, China) using the same primers. All newly generated sequences were deposited in the GenBank database (https://www.ncbi.nlm.nih.gov/genbank/; Table 1).

**Table 1.** Species name, strain numbers, and GenBank accession numbers for the phylogenetic analyses performed in the present study. Entries in bold were newly generated for this study.

Species name	Strain number	Locality	GenBank accession number	
			ITS	LSU D1/D2
*Vishniacozymaalagoana*	CBS 15966^T^	Brazil	MH885328	MH909005
*Vishniacozymacarnescens*	CBS 973^T^	Italy	NR_130695	NG_058430
*Vishniacozymacatalpae*	CGMCC 2.6902^T^	China	OP470302	OP470206
*Vishniacozymachanghuana*	CBS 16556^T^	Taiwan	NR_182838	MT906468
*Vishniacozymadimennae*	CBS 5770^T^	New Zealand	NR_144808	NG_058431
***Vishniacozymadiospyri***	**NYNU 221044^T^**	**China**	**OP954624**	**OP954569**
***Vishniacozymadiospyri***	**NYNU 2211329**	**China**	**PQ496711**	**PQ496709**
*Vishniacozymaellesmerensis*	JCM 32573^T^	Canadian Arctic	NR_173768	LC335797
***Vishniacozymaeriobotryae***	**NYNU 229203^T^**	**China**	**OP566897**	**OP566895**
***Vishniacozymaeriobotryae***	**NYNU 229144**	**China**	**PP580371**	**PP580369**
*Vishniacozymaeuropaea*	CGMCC 2.3099^T^	China	NR_174757	MK050335
*Vishniacozymafloricola*	NCAIM Y.02320^T^	Hungary	PP337022	PP261370
*Vishniacozymafoliicola*	CGMCC 2.2471^T^	China	NR_144809	NG_067769
*Vishniacozymaglobispora*	CBS 6981^T^	Canada	NR_073235	NG_070507
***Vishniacozymaguiyangensis***	**NYNU 22831^T^**	**China**	**OP566869**	**OP566870**
***Vishniacozymaguiyangensis***	**NYNU 236231**	**China**	**PP580372**	**PP580370**
***Vishniacozymaguiyangensis***	**NYNU 236232**	**China**	**PP580374**	**PP580373**
*Vishniacozymaheimaeyensis*	CBS 8933^T^	Iceland	NR_077070	NG_058432
*Vishniacozymainsularis*	BRIP 28256^T^	Australia	NR_175761	–
*Vishniacozymakurtzmanii*	CBS 12229^T^	USA	NR_168771	FR820582
*Vishniacozymamarinae*	CGMCC 2.6837^T^	China	OP470294	OP470198
*Vishniacozymamelezitolytica*	CGMCC 2.3472^T^	China	NR_174755	MK050330
*Vishniacozymanebularis*	CBS 12283^T^	Taiwan	–	EU266921
*Vishniacozymaparavictoriae*	CGMCC 2.6918^T^	China	OP470300	OP470204
*Vishniacozymapeneaus*	CBS 2409^T^	USA	NR_165987	AB035051
*Vishniacozymaphoenicis*	KBP Y-6564^T^	Russia	MN449981	MN449981
***Vishniacozymapingtangensis***	**NYNU 23281^T^**	**China**	**OQ851896**	**OQ851894**
***Vishniacozymapingtangensis***	**NYNU 236258**	**China**	**PQ496708**	**PQ496707**
*Vishniacozymapini*	CGMCC 2.6849^T^	China	OP470296	OP470200
*Vishniacozymapseudocarnescens*	CGMCC 2.6457^T^	China	OR077051	OR077057
*Vishniacozymapseudodimennae*	CGMCC 2.6790^T^	China	OM417179	OM417179
*Vishniacozymapseudopenaeus*	CGMCC 2.3165^T^	China	NR_174756	MK050333
*Vishniacozymapsychrotolerans*	CBS 12690^T^	Portugal	NR_111656	JN193445
*Vishniacozymapyri*	CGMCC 2.6870^T^	China	OP470298	OP470202
*Vishniacozymasinopodophylli*	CGMCC 2.6857^T^	China	OP470297	OP470201
*Vishniacozymataibaiensis*	CBS 9919^T^	Taiwan	NR_144810	NG_058434
*Vishniacozymataiwanica*	BCRC 23477	Taiwan	NR_182839	MT906477
*Vishniacozymatephrensis*	CBS 8935^T^	Iceland	NR_144812	KX507032
*Vishniacozymaterrae*	KCTC 27988^T^	Korea	MZ734447	NG_241953
***Vishniacozymatianchiensis***	**NYNU 236163^T^**	**China**	**OR426458**	**OR426457**
***Vishniacozymatianchiensis***	**NYNU 2311236**	**China**	**PQ496716**	**PQ496715**
*Vishniacozymavictoriae*	CBS 8685^T^	Antarctica	NR_073260	AF363647
*Vishniacozymazhenxiongensis*	CGMCC 2.6901^T^	China	OP470301	OP470205
*Tremellaglobispora*	CBS 6972^T^	Canada	AF444432	AF189869
*Tremellaflava*	CBS 8471^T^	Taiwan	NR_155935	AF042221
*Tremellataiwanensis*	CBS 8479^T^	Taiwan	AF042412	AF042230
*Tremellaresupinata*	CBS 8488^T^	Taiwan	AF042421	AF042239
*Tremellabrasiliensis*	CBS 6966^T^	Costa Rica	AF444429	AF189864
*Apiotrichumporosum*	CBS 2040^T^	Germany	AF414694	AF189833

**^T,^** type strain.

### ﻿Phylogenetic analysis

Sequences from 49 strains were employed for phylogenetic analysis. *Apiotrichumporosum* Stautz, CBS2040, was chosen as the outgroup. Phylogenetic analyses were based on a combined ITS and LSU dataset. Aside from the newly generated sequences, additional related sequences were obtained from GenBank (Table 1). Sequences for the individual loci were aligned using MAFFT v.7.110 (Katoh and Standley 2013) with the G-INI-I option. Poorly aligned regions were removed and manually adjusted using MEGA v.11 (Tamura et al. 2021). PhyloSuite v.1.2.2 (Zhang et al. 2020) was employed to concatenate the aligned sequences of the different loci.

Maximum likelihood (ML) analyses were performed using RAxML v.8.2.3 (Stamatakis 2014) under a GTRGAMMA model with one thousand rapid bootstrap (BS) replicates. Bayesian inference (BI) analyses were conducted using MrBayes v.3.2.2 with a GTR + I + G model of DNA substitution and a gamma distribution rate variation across locations (Ronquist et al. 2012). Two independent runs were employed, and each run had four chains and began from random trees. Trees were sampled every 1000^th^ generation, and the first 25% of trees were removed, while the other 75% of trees were retained to construct a 50% majority consensus tree and calculated Bayesian posterior probabilities (BPP). Each tree was visualized with its BS and BPP bootstrap values using Figtree v.1.4.3 (Andrew 2016).

## ﻿Results

### ﻿Molecular phylogeny

**Figure 1.**
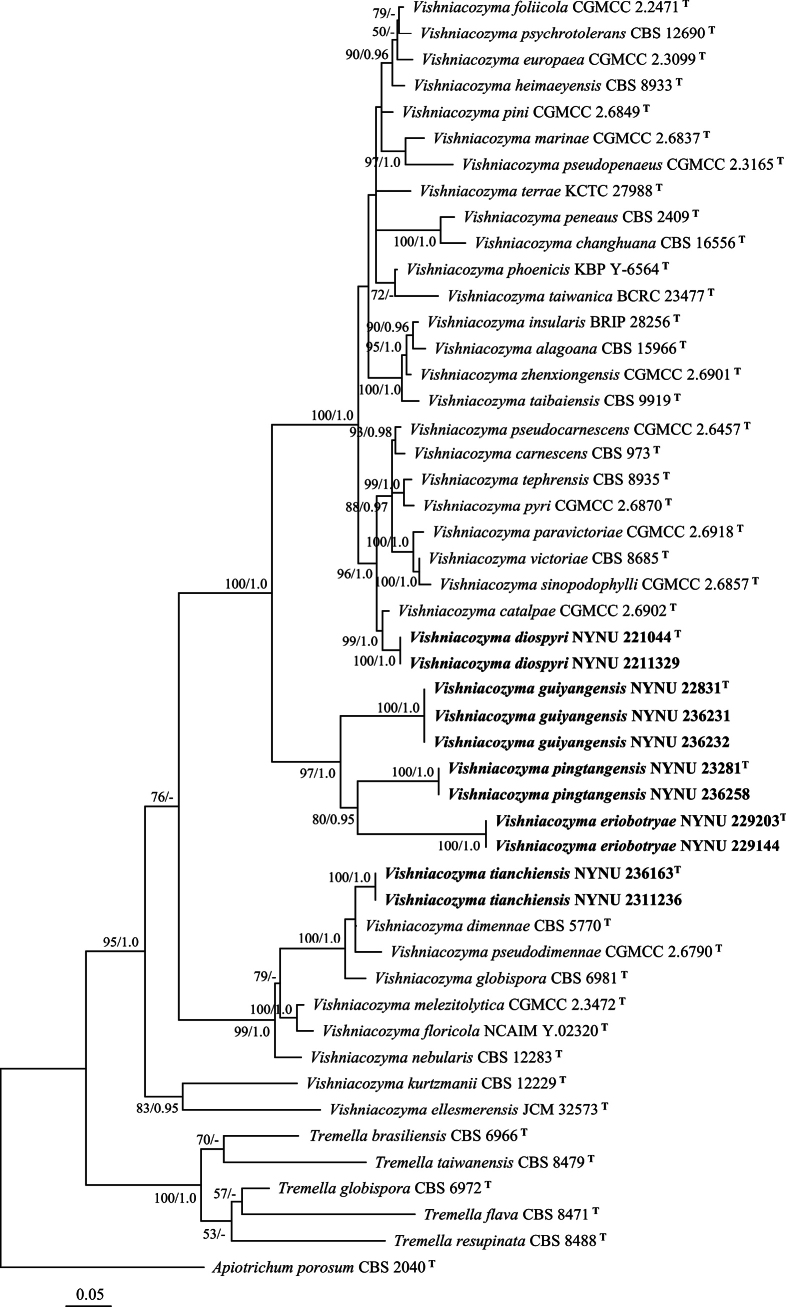
Maximum likelihood (ML) phylogenetic tree of *Vishniacozyma* founded on combined ITS and LSU sequence data. The tree was rooted with *Apiotrichumporosum* CBS 2040. The bootstrap values and Bayesian posterior probabilities over 50%/0.95 (BS/BPP) are indicated at the nodes. Sequences from type strains are marked with (T), and the new species are indicated in bold.

The total length of the concatenated dataset of two loci across the 49 samples was 1098 bp, including 493 bp for ITS and 605 bp for LSU. ML and BI methods generated similar topologies in main lineages, and only the topology generated by the ML method was presented along with BS values and BPP above 50% and 0.95, respectively, at the nodes (Fig. 1).

In the phylogenetic tree (Fig. 1), *Vishniacozyma* was monophyletic with well statistical support (BS/95, BPP/1.0). Eleven newly isolated strains formed five distinct and well-supported lineages distant from other *Vishniacozyma* species.

### ﻿Taxonomy

According to the phylogenetic and phenotypic analysis, five new species of *Vishniacozyma* are described.

#### ﻿ Vishniacozyma diospyri

Taxon classificationFungiTremellalesBulleribasidiaceae

C.Y. Chai & F.L. Hui sp. nov.

08B829EE-816E-5FC7-8238-C17CF1911645

853420

Figs 2A, F

##### Etymology.

The specific epithet *diospyri* refers to *Diospyros*, the name of the genus from which the type strain was isolated.

##### Typus.

China. • Henan Prov.: Neixiang Co., Baotianman Nature Reserve (33°29'07"N, 111°52'51"E), Sep 2022, J.Z. Li, in the phylloplane of *Diospyroslotus*, NYNU 221044 (holotype CICC 33574^T^, GenBank: OP954624, OP954569); culture ex-type PYCC 9955.

##### Description.

On YM agar after seven days at 20 °C, the streak culture is yellowish-cream, butyrous, and smooth, with an entire margin. After three days in YM broth at 20 °C, cells are ovoid, ellipsoidal, and cylindrical, 2.5–4.4 × 4.1–13.7 μm, and single; budding is polar. After one month at 20 °C, a ring and sediment are present. In Dalmau plate culture on CMA, pseudohyphae and hyphae are not formed. Sexual structures are not observed on PDA, CMA or V8 agar. On corn meal agar, ballistoconidia are not produced. Glucose fermentation is absent. Glucose, sucrose, raffinose, melibiose, galactose, lactose, trehalose, maltose, melezitose, methyl-α-D-glucoside, cellobiose, salicin (weak), L-sorbose, L-rhamnose, D-xylose, L-arabinose, D-arabinose, 5-keto-D-gluconate, D-ribose, glycerol (delayed), erythritol, ribitol (delayed), galactitol, D-mannitol, D-glucitol, myo-inositol, DL-lactate, succinate, D-gluconate, D-glucosamine, N-acetyl-D-glucosamine, 2-keto-D-gluconate, D-glucuronate, and glucono-1,5-lactone are assimilated as sole carbon sources. Inulin, methanol, and ethanol are not assimilated. Nitrite and L-lysine are assimilated as sole nitrogen sources. Nitrate, ethylamine, and cadaverine are not assimilated. Maximum growth temperature is 25 °C. Growth in vitamin-free medium is positive. Growth on 50% (w/w) glucose-yeast extract agar is negative. Starch-like substances are not produced. Urease activity is positive. Diazonium Blue B reaction is positive.

**Figure 2.**
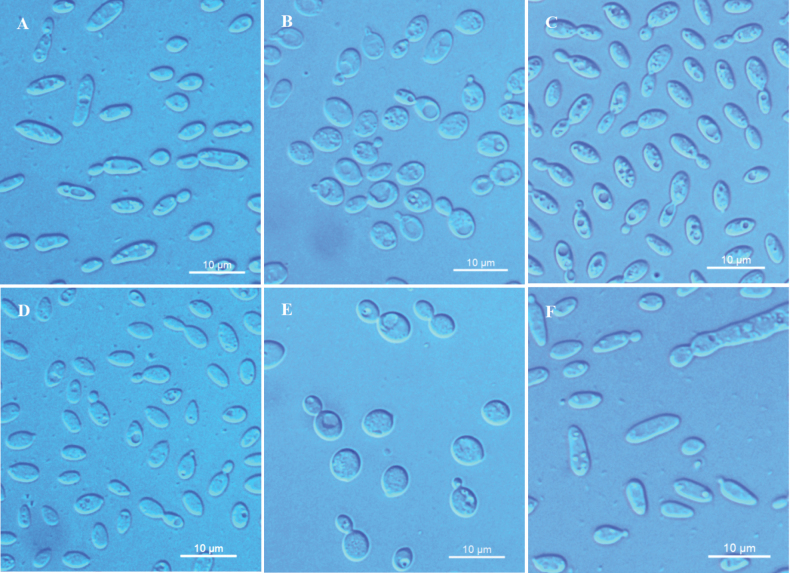
Budding cells of **A***Vishniacozymadiospyri* sp. nov. NYNU 221044^T^**B***V.guiyangensis* sp. nov. NYNU 22831^T^**C***V.pingtangensis* sp. nov. NYNU 23281^T^**D***V.eriobotryae* sp. nov. NYNU 229203^T^**E***V.tianchiensis* sp. nov. NYNU 236163^T^ following growth in YM broth for three days at 20 °C **F** Elongate budding cells of *V.diospyri* sp. nov. NYNU 221044^T^ following growth on CMA for seven days at 20 °C. Scale bars: 10 μm.

##### Additional strain examined.

China. • Henan Prov.: Neixiang Co., Baotianman Nature Reserve (33°29'07"N, 111°52'51"E), in the phylloplane of *Cornusofficinalis*, Sep 2022, J.Z. Li, NYNU 2211329 (GenBank: PQ496711, PQ496709).

##### Note.

In the phylogenetic analyses, *V.diospyri* formed a separate branch that clustered with *V.catalpae* with high support (BS/99, BPP/1.0; Fig. 1). However, *V.diospyri* differs from *V.catalpae* by 20 nucleotides (12/486 in ITS and 8/559 in LSU) and by its ability to assimilate D-arabinose, succinate, and D-glucosamine and its inability to assimilate inulin, ethylamine, and cadaverine.

#### ﻿ Vishniacozyma guiyangensis

Taxon classificationFungiTremellalesBulleribasidiaceae

C.Y. Chai & F.L. Hui sp. nov.

540E6514-466D-5D57-BDC0-067027C3737B

853421

Fig. 2B

##### Etymology.

The specific epithet *guiyangensis* refers to the geographic origin of the type strain, Guiyang city, Guizhou Province.

##### Typus.

China. • Guizhou Prov.: Guiyang City, Guiyang Medicinal Botanical Garden (26°34'51"N, 106°42'36"E), Aug 2022, in the phylloplane of *Distyliumracemosum*, L. Zhang & F.L. Hui, NYNU 22831 (holotype CICC 33569^T^, GenBank: OP566869, OP566870); culture ex-type PYCC 9934.

##### Description.

On YM agar after seven days at 20 °C, the streak culture is white-cream, mucoid, glistening, and smooth, with an entire margin. After three days in YM broth at 20 °C, cells are ovoid to ellipsoidal, 2.5–3.9 × 4.1–7.4 μm, and single; budding is polar. After one month at 20 °C, sediment is present. In Dalmau plate culture on CMA, pseudohyphae and hyphae are not formed. Sexual structures are not observed on PDA, CMA or V8 agar. On corn meal agar, ballistoconidia are not produced. Glucose fermentation is absent. Glucose, sucrose, raffinose, melibiose, galactose, lactose, trehalose, maltose, melezitose, methyl-α-D-glucoside, cellobiose, L-sorbose (delayed and weak), L-rhamnose, D-xylose, L-arabinose, D-ribose, glycerol, erythritol, galactitol (delayed), D-mannitol, D-glucitol, myo-inositol, DL-lactate, D-gluconate, D-glucosamine, N-acetyl-D-glucosamine, 2-keto-D-gluconate, and D-glucuronate are assimilated as sole carbon sources. Inulin, salicin, D-arabinose, 5-keto-D-gluconate, methanol, ethanol, ribitol, succinate, and glucono-1,5-lactone are not assimilated. Nitrate, nitrite, ethylamine, L-lysine, and cadaverine are not assimilated as sole nitrogen sources. Maximum growth temperature is 25 °C. Growth in vitamin-free medium is negative. Growth on 50% (w/w) glucose-yeast extract agar is positive. Starch-like substances are not produced. Urease activity is positive. Diazonium Blue B reaction is positive.

##### Additional strain examined.

China. • Henan Prov.: Songxian Co., Tianchi mountain (34°32'27"N, 112°16'39"E), Jun 2023, in the phylloplane of *Morusalba*, J.Z. Li, NYNU 236231 (GenBank: PP580372, PP580370), NYUN 236232 (GenBank: PP580374, PP580373).

##### Note.

In the phylogenetic analyses, three strains of *V.guiyangensis* clustered in a single clade with full support values (BS/100, BPP/1.0; Fig. 1), and *V.guiyangensis* was close to a clade formed by *V.pingtangensis* and *V.eriobotryae*, described in this study with significant support (BS/97, BPP/1.0; Fig. 1). *V.guiyangensis* differs from *V.pingtangensis* by 92 nucleotides (65/501 in ITS and 27/600 in LSU) and from *V.eriobotryae* by 64 nucleotides (17/596 in ITS and 47/596 in LSU). Physiologically, *V.guiyangensis* can be differentiated from these species by its inability to assimilate salicin, D-arabinose, 5-keto-D-gluconate, ribitol, succinate, and glucono-1,5-lactone. In addition, *V.guiyangensis* can grow on 50% (w/w) glucose-yeast extract agar, while the other two species cannot.

#### ﻿ Vishniacozyma pingtangensis

Taxon classificationFungiTremellalesBulleribasidiaceae

C.Y. Chai & F.L. Hui sp. nov.

B14FE6B2-7E5F-5DCF-A3FD-395300F72565

853422

Fig. 2C

##### Etymology.

The specific epithet *pingtangensis* refers to the geographic origin of the type strain, Pingtang County, Guizhou Province.

##### Typus.

China. • Guizhou Prov.: Pingtang Co., Sifangjing Vil. (25°7'45'N, 107°2'54"E), Feb 2023, in the phylloplane of *Acersaccharum*, D. Lu, NYNU 23281 (holotype CICC 33596^T^, GenBank: OQ851896, OQ851894); culture ex-type PYCC 9976.

##### Description.

On YM agar after seven days at 20 °C, the streak culture is white-cream, mucoid, glistening, and smooth, with an entire margin. After three days in YM broth at 20 °C, cells are ellipsoida, 2.4–3.7 × 3.7–6.4 μm, and single; budding is polar. After one month at 20 °C, a ring and sediment are present. In Dalmau plate culture on CMA, pseudohyphae and hyphae are not formed. Sexual structures are not observed on PDA, CMA or V8 agar. On corn meal agar, ballistoconidia are not produced. Glucose fermentation is absent. Glucose, sucrose, raffinose, melibiose, galactose, lactose, trehalose, maltose, melezitose, methyl-α-D-glucoside, cellobiose, salicin, L-sorbose (weak), L-rhamnose, D-xylose, L-arabinose, D-arabinose, 5-keto-D-gluconate, D-ribose, ethanol (weak), glycerol (delayed and weak), ribitol, galactitol, D-mannitol, D-glucitol, myo-inositol, DL-lactate (delayed), succinate (weak), D-gluconate, D-glucosamine (weak), N-acetyl-D-glucosamine, 2-keto-D-gluconate, D-glucuronate (weak), and glucono-1,5-lactone are assimilated as sole carbon sources. Inulin, methanol, and erythritol are not assimilated. Nitrate, nitrite (weak), ethylamine, L-lysine (weak), and cadaverine (delayed) are assimilated as sole nitrogen sources. Maximum growth temperature is 25 °C. Growth in vitamin-free medium is positive. Growth on 50% (w/w) glucose-yeast extract agar is negative. Starch-like substances are not produced. Urease activity is positive. Diazonium Blue B reaction is positive.

##### Additional strain examined.

China. • Henan Prov.: Songxian Co., Tianchi mountain (34°32'27"N, 112°16'39"E), Jun 2023, in the phylloplane of *Morusalba*, J.Z. Li, NYNU 236258 (GenBank: PQ496708, PQ496707).

##### Note.

In the phylogenetic analyses, *V.pingtangensis* was closely related to *V.eriobotryae*; both differ by 102 nucleotides (52/487 in ITS and 50/600 in LSU). Physiologically, they can be differentiated because *V.eriobotryae* has the ability to assimilate glycerol and the inability to assimilate inulin and erythritol, in addition to being able to grow at 30 °C, while *V.pingtangensis* cannot.

#### ﻿ Vishniacozyma eriobotryae

Taxon classificationFungiTremellalesBulleribasidiaceae

C.Y. Chai & F.L. Hui sp. nov.

CC5D35D4-10D4-5937-AF72-0890043E9BFF

853423

Fig. 2D

##### Etymology.

The specific epithet *eriobotryae* refers to *Eriobotrya*, the plant genus from which the type strain was isolated.

##### Typus.

China. • Guizhou Prov.: Guiyang City, Guiyang Medicinal Botanical Garden (26°34'51"N, 106°42'36"E), Aug 2022, in the phylloplane of *Eriobotryajaponica*, L. Zhang & F.L. Hui, NYNU 229203 (holotype GDMCC 2.312^T^, GenBank: OP566897, OP566895); culture ex-type PYCC 9940.

##### Description.

On YM agar after seven days at 20 °C, the streak culture is yellowish cream, mucoid, glistening, and smooth, with an entire margin. After three days in YM broth at 20 °C, cells are ovoid and ellipsoidal, 2.2–3.2 × 3.7–5.5 μm, and single; budding is polar. After one month at 20 °C, a ring and sediment are present. In Dalmau plate culture on CMA, pseudohyphae and hyphae are not formed. Sexual structures are not observed on PDA, CMA or V8 agar. On corn meal agar, ballistoconidia are not produced. Glucose fermentation is absent. Glucose, inulin (weak), sucrose, raffinose, melibiose, galactose, lactose, trehalose, maltose, melezitose, methyl-α-D-glucoside, cellobiose, salicin, L-sorbose (weak), L-rhamnose (weak), D-xylose, L-arabinose, D-arabinose, 5-keto-D-gluconate, ethanol (delayed and weak), erythritol, ribitol, galactitol, D-mannitol, D-glucitol, myo-inositol, DL-lactate (delayed and weak), succinate, D-gluconate, D-glucosamine (delayed and weak), N-acetyl-D-glucosamine, 2-keto-D-gluconate, D-glucuronate, and glucono-1,5-lactone are assimilated as sole carbon sources. Methanol and glycerol are not assimilated. Nitrate (delayed and weak), nitrite (delayed and weak), ethylamine (delayed and weak), and cadaverine (delayed and weak) are assimilated as sole nitrogen sources. Maximum growth temperature is 30 °C. Growth in vitamin-free medium is positive. Growth on 50% (w/w) glucose-yeast extract agar is negative. Starch-like substances are not produced. Urease activity is positive. Diazonium Blue B reaction is positive.

##### Additional strain examined.

China. • Guizhou Prov.: Guiyang City, Guiyang Medicinal Botanical Garden (26°34'51"N, 106°42'36"E), Aug 2022, in the phylloplane of *Buddlejadavidii*, L. Zhang & F.L. Hui, NYNU 229144 (GenBank: PP580371, PP580369).

##### Note.

*V.eriobotryae* assimilated nitrate as a sole source of nitrogen in a liquid medium, but not nitrite. If a yeast strain assimilates nitrate, it is expected to assimilate nitrite as well (Kurtzman et al. 2011). Nitrite is rather toxic, therefore, the auxanographic method was used to test the ability to utilize nitrite. On solid media, *V.eriobotryae* can weakly assimilate nitrate as a sole nitrogen source.

#### ﻿ Vishniacozyma tianchiensis

Taxon classificationFungiTremellalesBulleribasidiaceae

C.Y. Chai & F.L. Hui sp. nov.

55F3E740-3FB4-5A02-8BC4-31341A8B58F1

853424

Fig. 2E

##### Etymology.

The specific epithet *tianchiensis* refers to the geographic origin of the type strain, Tianchi Mountain, Songxian County, Henan Province.

##### Typus.

China. • Henan Prov.: Songxian Co., Tianchi mountain (34°32'27"N, 112°16'39"E), Jun 2023, in the phylloplane of *Salixmatsudana*, J.Z. Li, NYNU 236163 (holotype CICC 33617^T^, GenBank: OR426458, OR426457); culture ex-type PYCC 9988.

##### Description.

On YM agar after seven days at 20 °C, the streak culture is yellowish cream, mucoid, glistening, and smooth, with an entire margin. After three days in YM broth at 20 °C, cells are globose, 3.7–5.4 × 4–6.4 μm, and single; budding is polar. After one month at 20 °C, a ring and sediment are present. In Dalmau plate culture on CMA, pseudohyphae and hyphae are not formed. Sexual structures are not observed on PDA, CMA or V8 agar. On corn meal agar, ballistoconidia are not produced. Glucose fermentation is absent. Glucose, inulin, sucrose, raffinose, melibiose, galactose, lactose, trehalose, maltose, methyl-α-D-glucoside (delayed), cellobiose, salicin (weak), L-sorbose, L-rhamnose, D-xylose, L-arabinose, D-arabinose (weak), 5-keto-D-gluconate, D-ribose, glycerol (weak), erythritol, ribitol, galactitol, D-mannitol, D-glucitol, myo-inositol, DL-lactate, succinate, D-gluconate, D-glucosamine (delayed and weak), N-acetyl-D-glucosamine (weak), 2-keto-D-gluconate, D-glucuronate, and glucono-1,5-lactone are assimilated as sole carbon sources. Melezitose, methanol, ethanol, and citrate are not assimilated. Nitrate, nitrite, ethylamine, L-lysine, and cadaverine (weak) are assimilated as sole nitrogen sources. Maximum growth temperature is 25 °C. Growth in vitamin-free medium is positive. Growth on 50% (w/w) glucose-yeast extract agar is positive. Starch-like substances are not produced. Urease activity is positive. Diazonium Blue B reaction is positive.

##### Additional strain examined.

China. Henan Prov.: Xixia Co., Funiu Mountain (33°20'45"N, 111°47'37"E), Oct 2023, in the phylloplane of *Diospyroslotus*, S. Liu & Y.Z. Qiao, NYNU 2311236 (GenBank: PQ496716, PQ496715).

##### Note.

In the phylogenetic analyses, *V.tianchiensis* was grouped with *V.dimennae* (Fell & Phaff) X.Z. Liu, F.Y. Bai, M. Groenew. & Boekhout, *V.globispora* (B.N. Johri & Bandoni) X.Z. Liu, F.Y. Bai, M. Groenew. & Boekhout, and *V.pseudodimennae* in a clade with high support values (BS /100, BPP /1.0; Fig. 1). *V.tianchiensis* differs from *V.dimennae* by 21 nucleotides (14/479 in ITS and 7/596 in LSU), from *V.globispora* by 40 nucleotides (28/478 in ITS and 12/596 in LSU), and from *V.pseudodimennae* by 37 nucleotides (22/437 in ITS and 15/566 in LSU). Physiologically, *V.tianchiensis* can be differentiated from three closest known species, by the ability to assimilate inulin, melibiose, methyl-α-D-glucoside, and erythritol and the inability to grow at 30 °C.

## ﻿Discussion

The *Vishniacozyma* species share several phenotypic similarities, making it difficult to classify them accurately using phenotypic data alone. In this study, five new species were identified in *Vishniacozyma* according to the polyphasic approach recommended by Li et al. (2020) and Zhu et al. (2023). Based on both phenotypic and phylogenetic analyses, our study identified five novel species in which only asexual morphs were found. *V.diospyri*, *V.guiyangensis*, *V.pingtangensis*, *V.eriobotryae*, and *V.tianchiensis* are morphologically similar to their sister taxa, with significant differences regarding molecular data and physiochemical features.

Of all reported *Vishniacozyma* species, over 50% are linked to plant materials, of which approximately half are from plant leaves. The five new species described in this paper were also isolated from the surface of plant leaves, further enriching the diversity of the phylloplane fungi. However, considering their low abundance and rare occurrence, they could be classified as transient species. More importantly, most of these transient species colonized in plants, giving us an apparent indication of their role in plant growth in arid areas (Wei et al. 2022). For example, *V.pseudodimennae* and *V.victoriae* are transient species that have been frequently found in plants inhabiting dry environments (Buzzini et al. 2018; Wei et al. 2022), implying that these species may help plants survive in these areas. Similarly, five new *Vishniacozyma* species here described, *V.diospyri*, *V.guiyangensis*, *V.pingtangensis*, *V.eriobotryae*, and *V.tianchiensis*, were also isolated from plants. It is possible that these new species provide similar ecological roles as do *V.pseudodimennae* and *V.victoriae*.

To date, studies on *Vishniacozyma* have primarily focused on their taxonomy and ecosystem function (Chang et al. 2019; Nimsi et al. 2023). Consequently, interest in these fungi is not limited to their biodiversity and ecological role but encompasses potential agricultural, industrial, and medical uses with associated economic value (Bilański and Kowalski 2022; Nimsi et al. 2023).

## Supplementary Material

XML Treatment for Vishniacozyma diospyri

XML Treatment for Vishniacozyma guiyangensis

XML Treatment for Vishniacozyma pingtangensis

XML Treatment for Vishniacozyma eriobotryae

XML Treatment for Vishniacozyma tianchiensis

## ﻿Additional information

### Author ORCIDs

Shan Liu https://orcid.org/0009-0003-2845-1495

Dan-Yang Cai https://orcid.org/0009-0002-6866-7322

Chun-Yue Chai https://orcid.org/0000-0003-0284-5560

Feng-Li Hui https://orcid.org/0000-0001-7928-3055

### Data availability

All of the data that support the findings of this study are available in the main text.
